# Mechanical Parameters of Leather in Relation to Technological Processing of the Footwear Uppers

**DOI:** 10.3390/ma15155107

**Published:** 2022-07-22

**Authors:** Aura Mihai, Arina Seul, Antonela Curteza, Mariana Costea

**Affiliations:** Faculty of Industrial Design and Business Management, “Gheorghe Asachi” Technical University of Iasi, 700050 Iasi, Romania; aura.mihai@academic.tuiasi.ro (A.M.); antonela.curteza@academic.tuiasi.ro (A.C.); mariana.costea@academic.tuiasi.ro (M.C.)

**Keywords:** tensile test, leather, knitted fabric, footwear, Young’s modulus, Poisson’s ratio

## Abstract

This paper aimed to define two critical mechanical properties of leather—Young’s modulus and Poisson ratio—essential to the virtual simulation of the behaviour of the footwear uppers against the manufacturing operations of stitching and perforating. The following technological aspects were considered to analyse the materials from manufacturing conditions point of view: the number of layers (one layer and two layers), the nature of the materials used for uppers subgroups (calfskin-outer upper, sheep leather-lining, polyester knitted fabric-lining), the overlapping width in the stitching area, the number of parallel stitches (single stitch and double stitch), the punching interval and the type of perforations (simple and with eyelets), resulting in nine kinds of samples. Furthermore, the elasticity (Young’s modulus) and lateral contraction (Poisson’s ratio) were calculated during the tensile strength analysis performed on the SATRA STM 466 equipment. Both mechanical parameters are essential to simulate the behaviour of the virtual footwear prototypes in various conditions.

## 1. Introduction

Shape and footwear structure and the type of materials play an essential role in the functionality of the product and the fulfilment of its comfort requirements. Furthermore, due to technological and scientific progress in various related industries, the materials used in the footwear industry have diversified significantly [1]. Footwear materials are selected based on their chemical, physical and mechanical properties, accompanied by well-established criteria, such as the destination and constructive type of shoe and stress to which these materials are subjected, both in the manufacturing and wearing process. Nowadays, the virtual simulation of footwear behaviour has become an important tool in evaluating its performance during walking or running [2,3,4,5,6,7,8]. Among the methods of virtual simulation, finite element analysis (FEA) is the most appreciated because it replicates the physical phenomena using model discretisation in small regions called finite elements. FEA process is divided into several stages, including modelling and editing the 3D geometry of the object, establishing materials properties and their assignment to the 3D model components, setting analysis conditions and parameters, solving the model, and analysing the results. In these types of analysis, the mechanical characteristics of the materials play a crucial role in performing a simulation as close as possible to the real conditions in which the footwear product is worn.

In the context of new trends for product development, such as customised design and manufacturing [9,10], digitalisation and computer-based predicting tools applied in various fields, including footwear [11], this study aimed to define the critical mechanical properties of leather that are essential to virtually simulate the behaviour of the footwear uppers against wearing conditions or during manufacturing operations, such as stitching and perforating. The mechanical behaviour of the material reflects its response or deformation concerning an applied load or force. The basic mechanical properties that describe a material are rigidity, strength and deformability [12]. To introduce the material in FEA, for example, the properties that determine how it mechanically behaves must be defined, namely [13]: the Young’s modulus (*E*) and Poisson’s ratio. Additionally, the density and shear modulus can also be defined.

Young’s modulus is determined from the tensile curve, representing a measure of the stiffness of an elastic material Equation (1).

(1) $$E=\frac{FL_{0}}{A\Delta L}\;\left[\frac{N}{mm^{2}}\right]$$

where: *E*—Young’s modulus; *F*—applied force; *A*—initial cross-sectional area; Δ*L*—the difference between the initial length and the length-at-break; *L*_0_—initial length.

Poisson’s ratio describes the lateral contraction of the material and is equal to the ratio between transverse and longitudinal deformation Equation (2).

(2) $$\frac{\varepsilon_{transversal}}{\varepsilon_{longitudinal}}=\frac{\Delta l/l_{0}}{\Delta L/L_{0}}$$

where: Δ*l*—the difference between the initial width and the width at breaking; *l*_0_—initial width; Δ*L*—the difference between the initial length and the length-at-break; *L*_0_—initial length.

While various studies described the mechanical behaviour of materials and combinations for the bottom components [10,14], the uppers in footwear structure are studied much less from this perspective.

In the literature, a fairly extensive database [11,15,16] describes the mechanical behaviour of materials used for the virtual simulation of the shoe’s bottom assembly. For example, Young’s modulus and Poisson’s ratio are accessible for materials such as sole leather, leatherboard, cork, double density polyurethane, polyurethane foam, polyisoprene, rubber, silicon elastomer, ABS, PVC, etc. These databases are used in FEA studies to evaluate the impact of bottom assembly characteristics, such as the hardness [17], thickness [18,19], material properties [20] and design [21,22,23] on the soles and midsoles performance from the point of view of plantar pressure distribution, shock absorption, bending, twisting, etc., as well as foot biomechanics and comfort.

On the other hand, the available data for simulating a shoe’s upper assembly are limited due to the large variety of materials and manufacturing operations applied to these materials. The entire footwear product, including uppers, is rarely evaluated using finite element analysis because of the difficulty of experimental control [24,25,26,27]. Ruperez et al. [28] described the mechanical behaviour of two types of calfskin for uppers by calculating Young’s modulus and Poisson’s ratio from the tensile test, intending to use these data to simulate the behaviour of the footwear. The regular samples were cut in parallel and perpendicular to the backbone from the shoulder, butt and belly areas. In other studies, the properties of upper assembly are considered homogeneous over the entire surface of the footwear’s upper [24,25,26,27,28,29,30]. However, footwear is a structural complex multilayer product, where its parts/components are organised into groups and subgroups. By varying the materials that are part of each layer, the physical and mechanical performance of the entire product can be optimised [31].

The shoe upper’s parts are subjected to various technological processing operations such as skiving, punching and stitching. The research highlights several studies investigating the role of processing operations and their influence on materials’ mechanical behaviour. Features such as diameter and punching interval were evaluated on sixteen types of leather used for car seats [32]. The research shows that both parameters affected the leather’s physical (mass, density, air permeability) and mechanical properties (tensile strength). Phebe et al. [33] analysed the influence of the mechanical properties of sheep leather for garments on the efficiency of the seam. Among various characteristics, the authors analysed the tensile strength, elongation at break, the initial force, and the initial modulus of elasticity. More recent studies investigated the effectiveness of several types of stitches on chrome-tanned leather samples by varying parameters such as density [34]. Additionally, the tensile strength values were evaluated by comparing the behaviour of overlapped stitched and non-stitched samples from synthetic materials with PVC film [35]. Thus, to analyse the behaviour of the materials against footwear manufacturing operations of stitching and perforating, the following technological aspects were considered: the number of layers and the nature of the materials used for uppers subgroups, the overlapping width in the stitching area, the number of stitches, the punching interval and the type of perforations (simple or with eyelets). Three testing methodologies are suggested: tensile-strength and elongation-at-break of unprocessed flexible, stitched and perforated materials. By performing these tests, Young’s modulus of elasticity and Poisson’s lateral contraction coefficient are calculated, as mentioned above. As such, the authors of this research contribute to the enlargement of the database with mechanical properties of footwear materials necessary for future FEA studies to simulate the behaviour of virtual footwear prototypes in the new paradigm of digitalisation and Industry 4.0.

## 2. Materials and Methods

### 2.1. Materials

The uppers are subjected to various mechanical and physical stresses, such as longitudinal and transversal elongation, stretching and compression, bending, moisture and internal perspiration. To deal with these stresses, the materials structuring the outer group of uppers must have specific properties, including elastic-plastic behaviour at tensile stresses, tensile and tear strength, high flexibility, abrasion resistance, hygienic, aesthetic and ecological properties. Both traditional materials, namely leathers, with thicknesses between 0.9 and 2.5 mm (box, suede, nubuck, patent), as well as textiles (fabrics, knitwear) and synthetic alternatives to leathers, can meet these specifications.

The lining, whose primary role is to protect the dorsal surface of the foot, must be made of materials that have hygienic properties and ensure resistance to wet and dry abrasion, resistance to stretching and the action of sweat. Among the materials that meet these requirements are various assortments of thin leathers (sheep, goat, pig) and textile materials (woven and knitted fabric, nonwovens).

Calfskin leather (M1) with a thickness of 1.2 ± 0.1 mm was chosen for uppers, as the most common material used in footwear manufacturing, while two types of materials are considered for linings:

- Synthetic knitted fabric lining (M2)—Belgian over knitting with a thickness of 0.6 mm, ribbed structure 1:1, with retained mesh, with a fluffy velour appearance, made of 100 TEX polyester (PES) thread;
- Leather lining (M3)—sheep leather tanned in chromium salts, with a thickness of 0.4 ± 0.1 mm.

The mechanical behaviour model was obtained from the tensile test, performed for three types of samples: unprocessed (S1), stitched (S2) and perforated (S3). For every kind of sample, one layer of material is considered, which includes calfskin (M1) and two layers of materials which include calfskin with PES knitted fabric lining (M1 + M2) and calfskin with sheep leather lining (M1 + M3). Additionally, the number of parallel stitches and the overlapping width were considered for the stitched samples. As a result, four types of samples have resulted: single-stitched seam with 6 mm (S2a) and 8 mm (S2b) overlapping, double-stitched seam with 6 mm (S2c) and 8 mm (S2d) overlapping. Furthermore, the shape of perforations and the punching interval were varied for perforated samples. Therefore, the following types of samples were obtained: simple perforations with 4 mm (S3a) and 8 mm (S3b) punching intervals and samples with eyelets with 4 mm (S3c) and 8 mm (S3d) punching intervals.

### 2.2. Design of Experiments

A design of experiment method was applied to calculate the optimal number of determinations needed to perform a conclusive test for all three types of samples (S1, S2, S3). The main objective of this statistical and mathematical tool is to maximise the quality of the results and minimise the resources needed [36,37,38]. The designs of experiment methods are varied and applied in various fields, from agriculture to electronics [36], being adapted according to the objective of the experiment, the number of variables and levels [39,40]. These include the method of factorial plans, the method of fractional plans, the method of Latin squares and the Taguchi method.

Since the purpose of this experiment was not to optimise a process but to identify the optimal number of experiments for each test, a complete factorial plan was selected because it allows the cross-sectional design of the experiment and considers two or more variables—each of them with several levels. Such a plan is symbolised by X^k^, where k is the number of variables and X is the number of levels. Factorial plans are optimal in industrial applications and can be adapted accordingly to determine the number of experiments needed to test the materials. When the number of variables increases, the principle of fractional planes is applied, which reduces the number of experiments by half. In the case of an X^k^ plane, the half-fractional experiment is of type X^k−1^. Therefore, nine experiments were performed for each type of sample.

### 2.3. Equipment and Preparatory Stages

Leather is an anisotropic material. Therefore, most studies consider samples’ orientation in experiments according to the two main directions, parallel and perpendicular to the animal’s backbone [41,42]. However, according to leather cutting practices in a footwear company, the following rule is observed in the manufacturing process: *the direction of maximum stress of the parts coincides with the direction of minimum elongation of the material*. Additionally, in any simulation process, ideally the real manufacturing conditions are reproduced as accurately as possible. Therefore, the sampling was taken longitudinally with the backbone, and consequently, the entire load was applied in the axial direction of the tested sample to reproduce the manufacturing conditions used by footwear producers.

The tests were performed using the SATRA STM 466 tensile testing machine (SATRA Technology Centre, Kettering, Northamptonshire, UK) with computer control and adjacent software, ensuring statistical analysis and data interpretation.

Before the tensile test, all samples were conditioned for 24 h at a temperature of 20 ± 20 °C and relative humidity of 65 ± 2%. The thickness of the materials was manually determined on each sample using a micrometre. All samples were tested until breaking with a lower clamp speed of 100 ± 25 mm/min. The distance between the clamps was set individually for each category of the samples (S1, S2, S3) according to their length.

Before starting the testing process, the initial settings were established: the units of measurement and the speed of movement of the clamp, the type of results and additional data about the initial thickness, length and width of the samples. The following test results were registered: maximum force (N); cross-section area (mm^2^); sample length and width (mm); and the Young’s modulus (N/mm^2^).

The elastic modulus of the materials is automatically calculated by SATRA STM 466 adjacent software. According to the equipment provider, the software application calculates the value of the elastic modulus based on Equation (1) considering the maximum elastic load from the tensile test. The change in the sample’s width was measured, and the Poisson’s ratio was calculated as shown in Equation (2). The measurement was manually performed before sample breaking. The ruler was fixed in the same position to limit possible errors.

### 2.4. Method

#### 2.4.1. Flexible Unprocessed Samples 

**Figure 1:**
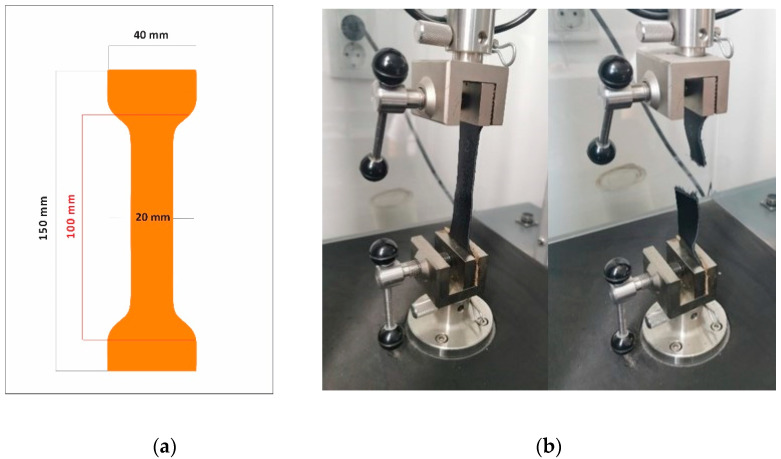
(**a**) Shape and dimensions of the unprocessed samples (S1); and (**b**) unprocessed sample position in the testing machine. Before and after testing.

The elasticity modulus *E* for unprocessed (S1) one-layer and two-layers samples was calculated using a tensile strength and elongation test based on ISO 3376:2020: Leather—Physical and mechanical tests—Determining tensile strength and percentage elongation [43]. The shape and dimensions of the samples are presented in Figure 1a. Nine experiments/tests were performed for each variable, resulting in 27 samples. Two-layer samples were joined by overlapping and catching the ends between the machine’s clamps, as shown in Figure 1b. The distance between the clamps was set to 100 mm.

#### 2.4.2. Stitched Samples 

The elasticity modulus *E* for the stitched (S2) one-layer and two-layers samples was calculated using an adapted methodology based on SATRA TM29 and ISO 17697:2003: Footwear—Test methods for uppers, lining and insocks—Seam strength [44].

**Figure 2:**
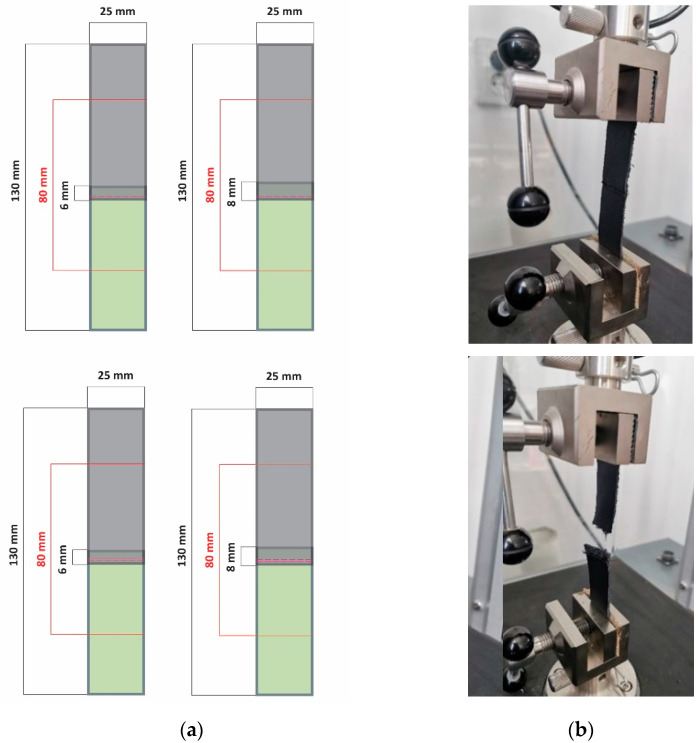
(**a**) Shape and dimensions of the stitched samples (S2 a, b, c, d); and (**b**) stitched sample position in the testing machine. Before and after testing.

Rectangular samples with the dimensions shown in Figure 2a were obtained by overlapping two strips of calfskin leather on 6- and 8-mm width and then sewn together using a single- or double-stitched seam. Previous to stitching, the overlapping area was skived. Stitching was performed on the postbed Global LP 9971 sewing machine (Global International B.V., Haarleem, The Netherlands), which makes a simple lock stitch seam using polyester thread number 50/3 with a 5 stitches/cm density. The selected needle has a twisted wedge (LR) section and a diameter of 0.9 mm. The distance between the two parallel stitches was 2 mm. The seam was reinforced at the ends to prevent slipping. The second layer was not stitched to reproduce the free lining in the footwear structure. The joining of the two-layers samples was performed by overlapping and clatching the ends of the samples between the machine’s clamps, as shown in Figure 2b. Nine experiments/tests were performed for each variable, resulting in 108 samples. The distance between the clamps was set to 80 mm. The thickness of the samples was manually measured in the overlapping area.

#### 2.4.3. Perforated Samples 

**Figure 3:**
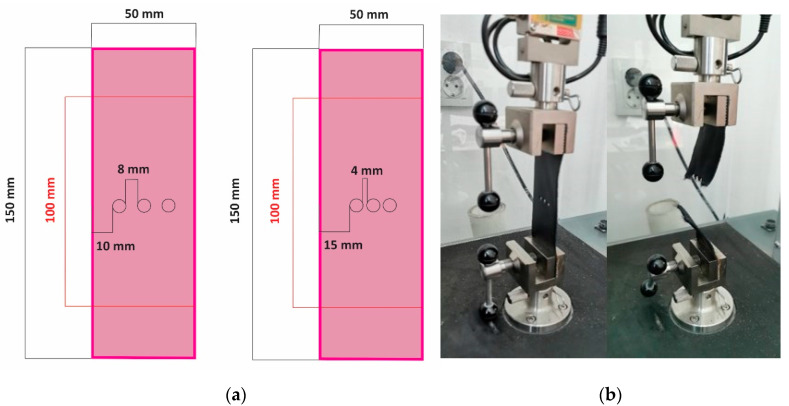
(**a**) Shape and dimensions of the perforated samples (S3); (**b**) perforated sample position in the testing machine. Before and after testing.

The elasticity modulus *E* for perforated (S3) one-layer and two-layers samples was calculated using an adapted methodology based on ISO 3376:2020: Leather—Physical and mechanical tests—Determining tensile strength and percentage elongation [43]. The perforations were positioned in parallel to the stress direction on the rectangular sample’s midline (Figure 3a) to reproduce the footwear lacing area.

Perforations were manually performed using a punch with a diameter of 5 mm. The two-layer samples were perforated together and caught using eyelets (S3c and S3d) or just overlapped (S3a and S3b) and clatched between the machine’s clamps (Figure 3b). Nine experiments/tests were performed for each variable, resulting in 108 samples. The distance between the clamps was set at 100 mm.

## 3. Results and Discussion

**Figure 4:**
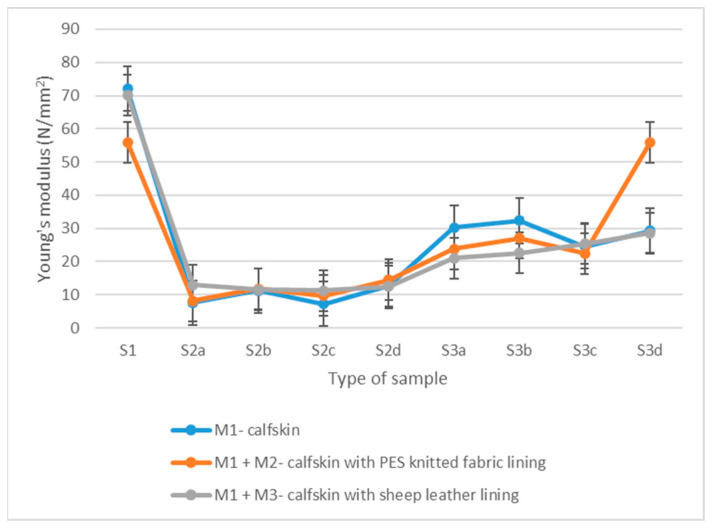
Young’s modulus mean values distribution.

**Figure 5:**
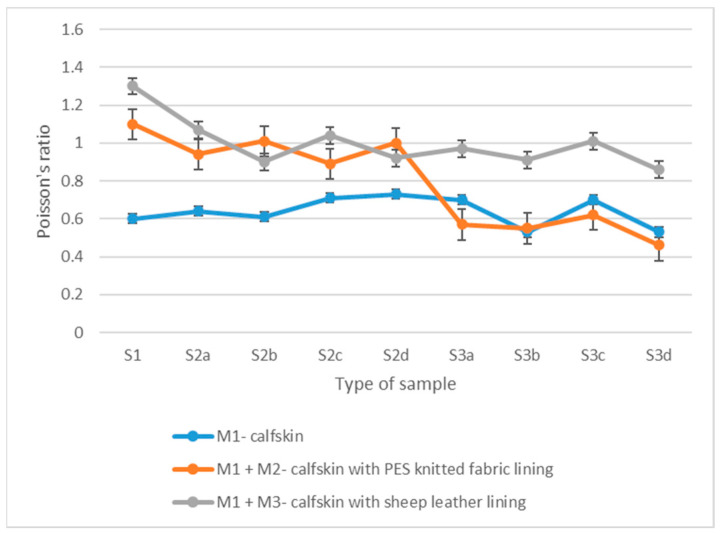
Poisson’s ratio mean values distribution.

The mean values and the coefficient of variation (CV) obtained for each sample type are listed in Table 1 for Young’s modulus and Table 2 for Poisson’s ratio. Figure 4 and Figure 5 show the graphical representation of the mean values of the Young’s modulus and Poisson’s ratio for each type of sample. 

**Figure 6:**
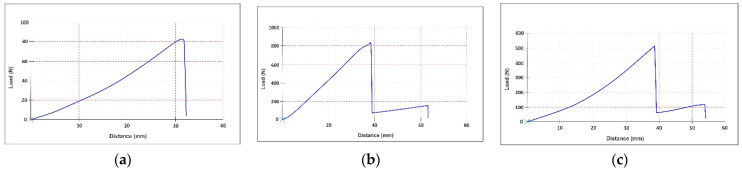
Tensile curve for unprocessed samples: (**a**) calfskin (M1); (**b**) calfskin with PES knitted fabric lining (M1 + M2); and (**c**) calfskin with sheep leather lining (M1 + M3).

**Figure 7:**
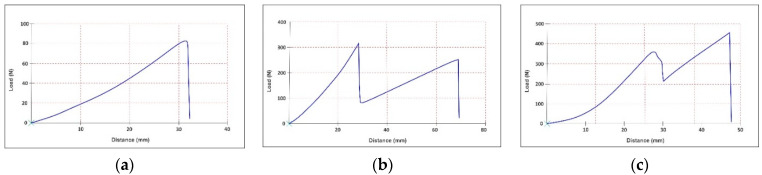
Tensile curve for stitched samples: (**a**) calfskin (M1); (**b**) calfskin with PES knitted fabric lining (M1 + M2); and (**c**) calfskin with sheep leather lining (M1 + M3).

**Figure 8:**
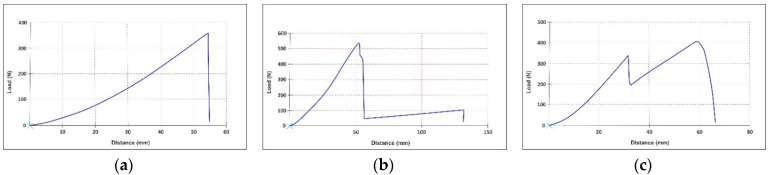
Tensile curve for perforated samples: (**a**) calfskin (M1); (**b**) calfskin with PES knitted fabric lining (M1 + M2); and (**c**) calfskin with sheep leather lining (M1 + M3).

The tensile curves are presented in Figure 6, Figure 7 and Figure 8. All the tensile curves show a complex behaviour of non-elastic, elastic and plastic-type, typical in the case of leather testing, which is a multilayer structure with different properties on the grain layer compared to the dermal layer. In addition, the coating film used to finish the leather also determines a particular behaviour. Thus, at the beginning of the curve, a first area is identified, characterised by a non-elastic behaviour, followed by a more stable area described by an elastic behaviour. As the stress increases, the first cracks appear on the leather’s surface (grain), and as these cracks amplify, a rupture occurs due to the failure of the dermal layer of the skin. The latter behaviour is, in fact, specific to plastics. Li Z. et al. observed the similar mechanical behaviour of natural cow leather in various tensile tests [42].

Comparable behaviour was found for all three categories of samples (unprocessed, stitched or perforated). In the case of doubled samples, either with PES-based fabrics (M1 + M2) or sheep leather (M1 + M3), the presence of the lining layer determines the appearance of the second moment of a break after the initial layer (M1) has yielded, a fact which corresponds to the reality of the practice of manufacturing footwear products. In the case of stitched samples, due to the seam’s thickness and the material’s perforation by the tip of the needle, the force-at-break is the lowest. These are followed by perforated samples, wherein holes are drilled but more significantly in diameter and sewn at greater distances. The best resistance is, of course, in the case of unprocessed samples. However, this situation appears quite rare in the practice of manufacturing because the design lines required are usually different from one model or another and require the structuring of the uppers in several parts by sewing or, as the case may be, the ornamentation of some parts by perforation. Otherwise, everyone would wear the same shoe model, with a uniformised design, in the same constructive type. These behaviours cause a significant reduction in the strength of uppers in the case of parts processed by sewing or perforation.

### 3.1. Flexible Unprocessed Samples

Leather has an extensive Young’s modulus range (from 20 to 100 N/mm^2^) which allows the elastic deformation of the material and its partial return to its original shape both during the wearing of the footwear and its manufacturing [28,45,46]. The obtained data show that the tested calfskin one-layer samples have a higher modulus of elasticity (72 N/mm^2^) compared to two-layer samples. *E* is reduced by approximately 3% (70.2 N/mm^2^) when a sheep leather lining is inserted and 22% (55.9 N/mm^2^) when PES knitted lining is used. In both situations, the break of the lining layer was produced later than the outer calfskin layer (Figure 6).

From the coefficient of variation point of view, Young’s modulus values are more variable for calfskin one-layer samples (29%) and two-layers samples with sheep leather lining (19.4%). However, these do not exceed 30%, concluding that the values are homogeneous. This distribution is explained by the natural origin of samples containing collagen fibres and varying the elastic properties over the entire surface [47]. Instead, a minor variation of values and a very homogeneous population is highlighted when a synthetic knitted lining with uniform structure properties is introduced (8.3%).

According to the literature, Poisson’s ratio values for most materials range between 0.3 and 0.5. However, it is found that leather can have values of this coefficient above the indicated limit, reaching 1.8, due to the porous structure and fibres, which may approach when the material is stretched in the longitudinal direction [47]. The results of the present study confirm this. The calfskin samples have a Poisson’s ratio of 0.6. When an additional layer is introduced into the structure, the coefficient significantly increases, namely by 83% in the case of the knitted lining (1.1) and 117% in the case of sheep leather lining (1.3). As the distribution of the coefficient of variation demonstrates, a very homogeneous distribution is observed in all three situations. However, similarly to the distribution of the Young’s modulus, natural materials, namely calfskin (10.8%) and calfskin with sheep leather lining (9.9%), have more dispersed values due to their structure.

The results for both Young’s modulus (72 N/mm^2^) and the Poisson’s ratio (0.6) for one-layer calfskin samples are very close to the data provided in the literature (69.9 N/mm^2^ and 0.7) [28,43], which highlights the accuracy of the performed tests and validates the obtained results.

### 3.2. Stitched Samples

Skiving the parts in the overlapping area and joining them by sewing which favoured the weakening of the ensemble of materials influenced the mechanical behaviour of the final product.

The stitched seam with 5 stitches/cm ensures uniform resistance of the sewn parts and a simultaneous break of the material and thread. All tested samples broke in the seam area.

Young’s modulus values decrease by approximately 80% compared to flexible unprocessed materials for the stitched samples, while the Poisson’s ratio is reduced by 15–20%.

As a general trend, it is highlighted that the Young’s modulus also increases by increasing the overlapping width and the number of parallel stitches. Comparing the results obtained between the stitched samples, smaller values for Young’s modulus could be seen for the samples with a single-stitched seam and 6 mm overlap. In comparison, the maximum values are emphasised in the case of samples with double stitch and 8 mm overlap.

These results are comparable to those reported in the specialised literature. For example, Phebe et al. [33] highlighted that sheepskin leather samples with a thickness of 0.6 mm, stitched using a thread with a fineness of 50, needle number 110 and a stitch of 3 stitches/cm density, have a mean initial modulus of elasticity equal to 4.4 N/mm^2^. On the other hand, the calfskin one-layer samples with 1.1 mm thickness, 6 mm overlapping and a single stitch tested in the present research have an average Young’s modulus equal to 7.6 N/mm^2^. Different degrees of fibres interlacing in the structure of sheepskins compared with calfskin leather explain the difference between these values (4.4 N/mm^2^ vs. 7.6 N/mm^2^).

Young’s modulus values increase once the lining is introduced into the structure of the samples. Sheep leather lining increases the analysed parameter by approximately 32%, while the synthetic knitted lining increases it by approximately 14%. The linings break later than calfskin in the case of the two-layer samples (Figure 7).

The analysed samples have a relatively heterogeneous character, with a medium and large spread. The maximum coefficient of variation is attributed to the calfskin samples with a double-stitched seam and 8 mm overlapping (41.8%). In contrast, two-layers samples with sheep leather lining, double stitch and 6 mm overlapping showed a medium spread of values (23.4%). The uneven elastic properties of natural materials [47], the type of skiving in overlapping areas, and the punching by needle cause a weakening of the material by 3–60%, depending on its origin. Additionally, the needle’s shape, the number of stitches and the width of the overlapping cumulative collaborate with this weakness [35]. 

The Poisson’s ratio values for stitched samples vary between 0.6 and 1.1, which is approximately 20% higher than unprocessed two-layer samples and approximately 18% lower than the one-layer calfskin samples. This value is because the stitch line acts as a constraint that reduces the lateral contraction for one-layer stitched samples, while the unstitched linings allow a more significant elongation.

Homogeneous distribution is observed in the case of calfskin samples with knitted lining. The coefficient of variation is below 15% due to the presence in the structure of synthetic material. However, for one-layer calfskin samples, the distribution of values is relatively heterogeneous (13.75–29.77%), while for two-layer samples with sheep leather lining, the distribution of values is heterogeneous (21.80–36.21%). Such a distribution is due to the irregular structure of the leather.

### 3.3. Perforated Samples 

Punching is a mechanical operation with a visible influence on the properties of the materials included in the structure of the footwear product.

According to the literature [32], the perforated calfskin leathers with a 5 mm punching interval show mechanical behaviour similar to unprocessed leathers. However, leather samples with a 3 mm punching interval show significant mass, bulk density, and flexibility changes, while those with a 2 mm punching interval change their shear strength behaviour. Therefore, a greater distance between perforations is recommended for the better resistance of the footwear uppers.

The Young’s modulus of perforated samples decreases by approximately 50% compared to unprocessed ones, while the Poisson’s ratio changes very little.

The material type primarily influences the studied parameters. The distance between the perforations and their type, act as secondary influencing parameters.

Young’s modulus decreases once the lining is introduced. Namely, the sheep leather lining reduces the elasticity modulus by approximately 16%, while the synthetic lining decreases it by approximately 14%.

As a general trend, it is highlighted that Young’s modulus also increases by an increasing punching interval. Moreover, the samples with a 4 mm punching interval have lower Young’s modulus values than those with an 8 mm punching interval.

The eyelets lead to a slight decrease in the average values of Young’s modulus for one-layer calfskin (20%) and two-layers with knitted lining (5%) compared to the simple perforated samples. However, the Young’s modulus increases for the two-layers samples with sheep leather by 36% under the same conditions.

From a variation point of view, Young’s modulus values are homogeneous (9–29.9%). The samples with a 4 mm punching interval show significantly higher variation than those with an 8 mm punching interval. This confirms that a shorter distance between the holes leads to more irregular mechanical behaviour of the material. A higher coefficient of variation is also identified for the samples with eyelets.

Poisson’s ratio values for perforated samples vary between 0.46 and 1.01. Two-layer samples with sheep leather lining have the lowest values for the Poisson’s ratio (0.46–0.52), followed by one-layer calfskin samples (0.53–0.70) and two-layers samples with synthetic knitted lining (0.86–1.01).

The lateral contraction for samples with an 8 mm punching interval is lower than samples with a 4 mm punching interval by approximately 20% for one-layer samples, 25% for two-layers samples with knitted lining and 7% for those with sheep leather lining.

The coefficient of variation for the Poisson’s ratio shows a minimum and medium distribution, defining the values as homogeneous.

## 4. Conclusions

Digitalisation, new business models, including virtual prototyping before physical samples are tested regarding their performance and manufacturability and the customisation of the product design are among the trends in the footwear sector worldwide. Thus, identifying the reliable mechanical properties of the materials after technological processing is critical for the virtual simulation of footwear performance.

In this study, the mechanical behaviour of unprocessed, stitched and perforated one-layer (calfskin) and two-layer (calfskin with sheep leather lining, calfskin with PES knitted fabric lining) samples was investigated using tensile strength and elongation at the break test. Furthermore, the elasticity modulus (Young’s modulus) and lateral contraction (Poisson’s ratio) were calculated from the tensile test to describe the behaviour of materials included in the structure of footwear’s uppers. In addition, the following technological aspects were taken into account to analyse the manufacturing behaviour of the materials: the number of layers and the nature of the materials used for the uppers subgroups, the overlapping width in the stitching area, the number of parallel stitches, the punching interval and the type of perforations (simple or with eyelets), resulting in nine types of samples. The statistical analysis of the experimentally obtained data shows that the values are representative.

Mean values’ analysis highlights that technological processing, such as stitching and punching, significantly reduces Young’s modulus values which change the behaviour of uppers during manufacturing and walking. Furthermore, this suggests the importance of correctly defining the materials’ properties in certain product areas, such as stitched and perforated ones. In existing studies [24,25,26,27,28,29,30], the properties are considered homogeneous over the entire surface of the footwear’s upper.

The analysis of Poisson’s ratio shows slight variations compared to Young’s modulus. However, it is found that, by inserting the natural leather lining, the Poisson’s ratio increases more compared to the synthetic knitted lining. Therefore, choosing materials with a lower value of the Poisson’s ratio is necessary for a more dimensionally stable structure.

The resulting mechanical behaviour model for all nine types of samples ensures a precise definition of the materials used in future work to simulate the performance of the virtual footwear product by modifying the design and technological parameters.

## Figures and Tables

**Table 1:** Young’s modulus mean values and coefficient of variation.

Type of Material		Type of Sample								
		S1	S2a	S2b	S2c	S2d	S3a	S3b	S3c	S3d
M1	Mean (N/mm^2^)	72	7.6	11.2	7.2	12.7	30.2	32.3	24.5	29.3
	CV (%)	29	23.8	27.8	51.2	34.9	18.7	12.3	27.9	22.3
M1 + M2	Mean (N/mm^2^)	55.9	8.2	11.8	9.7	14.5	23.8	27	22.5	55.9
	CV (%)	8.3	33.9	30.7	36.1	36.4	29.9	19.5	27.5	8.3
M1 + M3	Mean (N/mm^2^)	70.2	12.9	11.6	11.2	12.5	21	22.6	25.4	28.6
	CV (%)	19.4	35.2	38.4	23.4	43	16.5	9.0	21.3	19.9

M1—calfskin; M1 + M2—calfskin with PES knitted fabric lining; M1 + M3—calfskin with sheep leather lining. S1—unprocessed samples; S2a—single stitch with 6 mm overlapping; S2b—single stitch with 8 mm overlapping; S2c—double stitch with 6 mm overlapping; S2d—double stitch with 8 mm overlapping; S3a—simple perforations with 4 mm punching intervals; S3b—simple perforations with 8 mm punching intervals; S3c—samples with eyelets with 4 mm punching intervals; S3d—samples with eyelets with 8 mm punching intervals.

**Table 2:** Poisson’s ratio mean values and coefficient of variation.

Type of Material		Type of Sample								
		S1	S2a	S2b	S2c	S2d	S3a	S3b	S3c	S3d
M1	Mean (N/mm^2^)	0.60	0.64	0.61	0.71	0.73	0.70	0.53	0.70	0.53
	CV (%)	10.8	29.1	27.9	27.0	13.7	18.2	20.8	6.9	6.2
M1 + M2	Mean (N/mm^2^)	1.10	0.94	1.01	0.89	1.00	0.57	0.55	0.62	0.46
	CV (%)	4.4	5.3	4.4	4.7	3.2	3.9	2.7	20.5	5.8
M1 + M3	Mean (N/mm^2^)	1.30	1.07	0.9	1.04	0.92	0.97	0.91	1.01	0.86
	CV (%)	9.9	21.8	24.5	34.1	36.2	16.9	14.4	19.4	18.0

M1—calfskin; M1 + M2—calfskin with PES knitted fabric lining; M1 + M3—calfskin with sheep leather lining. S1—unprocessed samples; S2a—single stitch with 6 mm overlapping; S2b—single stitch with 8 mm overlapping; S2c—double stitch with 6 mm overlapping; S2d—double stitch with 8 mm overlapping; S3a—simple perforations with 4 mm punching intervals; S3b—simple perforations with 8 mm punching intervals; S3c—samples with eyelets with 4 mm punching intervals; S3d—samples with eyelets with 8 mm punching intervals.

## Data Availability

Not applicable.
